# Regarding: Using KOOS-PS to validate dichotomous global rating of improvement or worsening following total knee arthroplasty

**DOI:** 10.2340/17453674.2024.42743

**Published:** 2024-12-23

**Authors:** Siri B WINTHER, Anders SJØSTRØM, Sølvi LIABAKK-SELLI, Olav A FOSS, Tina S WIK, Jomar KLAKSVIK

**Affiliations:** 1 Department of Orthopedic Surgery, St. Olavs Hospital HF, Trondheim; 2 Department of Neuromedicine and Movement Science, Faculty of Medicine and Health Science, Norwegian University of Science and Technology NTNU, Trondheim, Norway

*Sir*,—We thank Riddle and Dumenci for their interest and comments on our agreement study “Can KOOS-PS be replaced with a simple anchor question in patients after TKA?: an agreement study of 2,478 primary surgeries” [1] evaluating a single anchor question as a substitute for KOOS-PS in assessing change in knee function.

Riddle and Dumenci raise several objections related to the choice of variables and data handling in our article [2]. They argue that dichotomizing variables introduces errors and that global ratings are associated with recall bias. They also state that the extent of chance-corrected agreement should have been reported. In their opinion, the KOOS-PS is not considered an acceptable gold standard for judging meaningful change following TKA. Based on these objections, Riddle and Dumenci call into question the article’s conclusion.

Riddle and Dumenci point out that dichotomization of variables leads to a loss of information and adds error. We agree that data reduction entails lower statistical precision, which could introduce errors if important details are lost in the process. However, we also see dichotomization as a useful tradeoff in certain contexts, prioritizing simplicity and interpretability. We acknowledge that the issue of information loss related to dichotomization could have been better addressed in the article.

Riddle and Dumenci state that global ratings are vulnerable to recall bias. We completely agree, and this is precisely why recall bias was highlighted as a key element of uncertainty in the background section of our article.

As expected, the vast majority of patients experience improved knee function following surgery. This implies an imbalance in data distribution, as shown in the presented cross-table. Riddle and Dumenci call for a chance-corrected agreement, as much of the observed agreement could be attributed to chance alone, and refer to their performed Kappa analysis. According to the kappa value, the level of agreement falls in the “fair” range. This information aligns with the already presented specificity of 27% and a negative predicted value of 52% in our manuscript, an indication of low test performance for patients with “worsened” joint function as stated in our conclusion. However, we do acknowledge that presenting expected agreement in addition to observed agreement could add valuable information to the article.

Riddle and Dumenci consider KOOS-PS not acceptable as a gold standard for judging meaningful change following TKA. From our clinical perspective, the aim of the study was to evaluate whether a single anchor question could serve as a substitute for KOOS-PS in assessing change in knee function. Therefore, whether there are defined gold standards or other variables besides KOOS-PS that are considered better for judging meaningful change is of little relevance to the topic of this article.

Riddle and Dumenci challenge the conclusion based on the objections mentioned above. However, it is unclear whether they disagree with the entire 3-part conclusion or just the first part. Based on the referred quote, we assume it to be the latter. We will try to explain the full rationale behind the conclusion, which is composed of the following 3 parts:

1. *“The analysis showed a high agreement between the anchor question and the KOOS-PS….”*
2. *“However, the KOOS-PS might be a valuable supplement in patients reporting worsened anchor….”*
3. *“The patient’s response on the anchor question is influenced by the level of pain at 1 year.”*

The first part of the conclusion is based on the overall agreement between the anchor question and KOOS-PS, indicating that agreement between the dichotomized variables is 11 times more likely than disagreement. The second part of the conclusion focuses on a smaller subgroup of dissatisfied patients, offering a more nuanced view of the results, as the overall agreement was significantly influenced by the high proportion of patients with improved function. The third part of the conclusion is based on observations of pain scores, where dissatisfied patients were associated with higher pain levels at the time of scoring. Thus, pain represents both a source of error and an explanation for why some patients with improved KOOS-PS are still dissatisfied.

For use in our clinical practice, a simple anchor question would be sufficient to estimate the proportion of patients with improved knee function after primary TKA. However, it would not be sufficient to identify patients with worsened knee function.
